# Prevalence and Associated Risk Factors of Urinary Schistosomiasis among Primary School Pupils in the Jidawa and Zobiya Communities of Jigawa State, Nigeria

**DOI:** 10.5334/aogh.3704

**Published:** 2022-08-16

**Authors:** J. B. Balogun, B. Adewale, S. U. Balogun, A. Lawan, I. S. Haladu, M. M. Dogara, A. U. Aminu, C. R. Caffrey, H. P. De Koning, Y. Watanabe, E. O. Balogun

**Affiliations:** 1 Department of Biological Sciences, Federal University Dutse, P.M.B. 7156, Jigawa State, NG; 2 Department of Public Health and Epidemiology, Nigerian Institute for Medical Research (NIMR), Lagos State, NG; 3 Department of Human Anatomy, College of Basic Medical Sciences, Federal University Dutse, P.M.B. 7156, Jigawa State, NG; 4 Jigawa State Ministry of Health, Block B, New Complex Secretariat, Takur Dutse, Jigawa State, NG; 5 Center for Discovery and Innovation in Parasitic Diseases, Skaggs School of Pharmacy and Pharmaceutical Sciences, University of California San Diego, 9500 Gilman Drive, La Jolla, CA 92093, USA; 6 Institute of Infection, Immunity and Inflammation, College of Medical, Veterinary and Life Sciences, University of Glasgow, Glasgow G12 8TA, UK; 7 Department of Biomedical Chemistry, Graduate School of Medicine, The University of Tokyo, Tokyo 113-0033, JP; 8 Department of Biochemistry, Ahmadu Bello University, Zaria 2222, Kaduna State, NG

**Keywords:** Prevalence, Risk factors, Pupils, Schistosomiasis, Schistosoma eggs

## Abstract

**Background:**

Urogenital schistosomiasis (UgS) is a parasitic disease caused by *Schistosoma haematobium* and can lead to chronic ill-health. Nigeria is endemic for schistosomiasis, but epidemiology of UgS has not been studied in most states. This study was conceived with the aim to contribute towards an accurate national picture of UgS in Nigeria. The prevalence of UgS and the associated risk factors were for the first time investigated among primary school pupils in Jidawa and Zobiya communities of the Dutse Local Government Area (LGAs) of Jigawa State, Nigeria.

**Method:**

Focus group discussions with teachers and parents were conducted. After obtaining written consent from parents, questionnaires were administered to pupils to obtain socio-demographic data and information on water contact activities. Urine samples (279) were collected and processed by the urine filtration technique to evaluate haematuria and the presence of *S. haematobium* eggs.

**Results:**

Prevalences of 65.7% (90/137) and 69.0% (98/142) were recorded in the Jidawa and Zobiya communities, respectively. In both communities, there was a significant association between gender and UgS: 63.3% of the infected pupils were males as compared to 36.7% females (χ^2^ = 5.42, p = 0.020). Grade 5 students had a significantly higher prevalence (χ^2^ = 17.919, p = 0.001) (80.0%) compared to those in grades 2, 3, 4, and 6 (63.8%, 66.7%, 61.5%, and 64.6%, respectively). Water contact activities showed that pupils involved in fishing, irrigation, and swimming were at greater risk of becoming infected in Jidawa and Zobiya, with odds ratios (risk factors) of 5.4 (0.994–28.862) and 4.1 (1.709–9.862), respectively (p = 0.05).

**Conclusion:**

Both the Jidawa and Zobiya communities of the Dutse LGAs of Jigawa State are hyperendemic for UgS. In collaboration with the State Ministry of Health, mass administration of praziquantel was carried out in the Jidawa and Zobiya communities after this study.

## Introduction

Schistosomiasis is a chronic and debilitating neglected tropical disease that is caused by water-borne digenetic trematodes of the genus *Schistosoma*. The five medically important species are *Schisotosoma haematobium, S. mansoni, S. japonicum, S. mekongi, S. guineensis*, and *S. intercalatum* [1,2]. The first three are the most relevant from a global health perspective. Adult male and female schistosomes reside in the blood system, specifically the vesical venous plexus of the bladder (*S. haematobium*) or mesenteric vessels of the gastrointestinal tract (*S. mansoni* and *S. japonicum*). At these sites, mated female worms lay eggs, which are expelled via urine or feces, respectively, into the environment [3,4]. In freshwater, the eggs hatch to release miracidia, which seek out and penetrate intermediate snail hosts. In the appropriate host, asexual reproduction leads to the development of sporoscysts and their terminal differentiation as cercariae. Cercariae are released back into the water in response to sunlight and penetrate human skin to establish themselves in the systemic circulation as schistosomula. These mature in the liver into adult worms that end up in the blood vessels around the bladder or intestines to continue the cycle [4,5,6].

Whereas *S. haematobium* is responsible for urogenital schistosomiasis (UgS), the other two species cause intestinal schistosomiasis (IS). Both forms of the disease can lead to chronic and morbid ill-health that may lead to low self-esteem and stigmatization. UgS is associated with haematuria in children and adults, with haematospermia and painful ejaculation in adult males, and with abnormal vaginal discharges and pelvic discomfort in females [6,7,8,9,10,11].

UgS and IS are major public health problems affecting over 230 million people living in 54 tropical and sub-tropical countries, mostly in rural regions [8,12,13], with an estimated cost of 1.4 million disability-adjusted life years (DALYs) [14]. Schistosomiasis in females, known as female genital schistosomiasis (FGS) or gynaecological schistosomiasis, is gaining particular attention due to evidence linking FGS with increased risk of HIV acquisition and the development of cervical cancer [15], showing FGS to be an emerging sexual and reproductive health problem.

With over 25 million people infected, Nigeria has the highest endemicity for schistosomiasis in the world, and UgS is the leading form of the disease [16,17]. Schistosomiasis was first documented in any part of modern-day Nigeria in 1881 in the old Borno Empire, which is comprised of regions that form the present Borno State in North-Eastern Nigeria [18] and has since been shown to be widespread across the country, although prevalence varies by district [19]. In spite of mass chemotherapeutic programs with praziquantel (PZQ) that are aimed at reducing morbidity, the prevalence of the disease in Nigeria may be increasing due to poverty, poor public health infrastrucutres, and low literacy levels [20]. For many communities, including for Jigawa State, there is little information on the prevalence or incidence of the disease [19], including for high-risk school-age children, and this is a serious hindrance to control efforts. Accordingly, this study was designed to investigate the prevalence and risk factors associated with UgS among primary school pupils in Jidawa and Zobiya, which are rural communities in Dutse Local Government Area of Jigawa State, Nigeria, which have not been previously investigated for schistosomiasis. In addition, these communities have a large earth dam used for agricultural purposes including irrigation, fishing, and the provision of drinking water for livestock, hence, the choice of Jidawa and Zobiya for this study. Furthermore, the dam water is used by locals for bathing and other domestic purposes, putting them at high risk of being infected by any water-borne pathogens present in the reservoir. Reports abound on the contribution of agricultural dams to high prevalence of schistosomiasis [21,22]. This study provides the first dataset on the prevalence and the risk factors of UgS in Jigawa State and serves as baseline data for planing schistosomiasis control programs according to the World Health Organization (WHO) recommendations.

## Materials and Method

### Consent and ethics

Ethical clearance was granted by the Ethics Committee of the Federal University, Dutse, Jigawa State, Nigeria. In addition, approval for the study was obtained from the Department for Neglected Tropical Diseases of Jigawa State Ministry of Health. Stakeholders at the study communities, including community heads, local Department for Health officials, teachers, parents, and community residents, were adequately informed about schistosomiasis and the purpose of the study via health outreach and sensitization visits to the communities by our team.

### Study area

**Figure 1:**
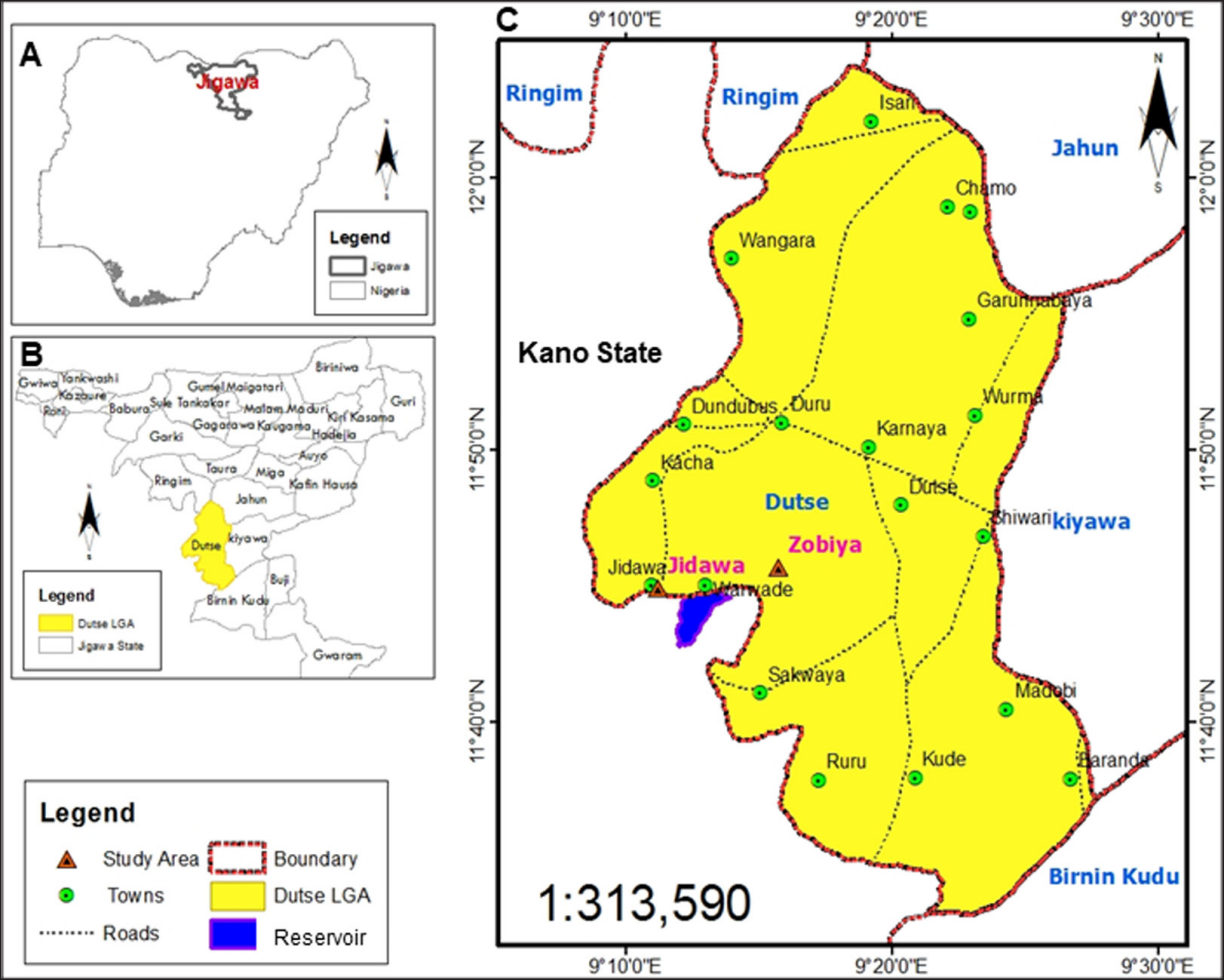
Map of the study locations (Source: GIS and Remote Sensing Software Arc Map 10.1 Department of Geography ABU, Zaria). **(A)** Map of Nigeria showing Jagawa State, a state in Northern Nigeria, which is has been identified as one of the few states lacking data on the prevalence of schistosomiasis [19]. **(B)** Detailed map of Jigawa State showing the local government areas (LGAs). The study locations are communities in Dutse LGA (yellow colour). **(C)** The expanded map of Dutse LGA showing the study area. The study communities were Jidawa and Zobiya, which are approximately 2 km apart and both walking distances from Warwade dam that is in between the communities. Warwade dam is a water body used by the locals for recreational, fishing, and agricultural purposes, and it was recently reported to be heavily infested with snails that transmit UgS [24].

The study was conducted in two communities, Jidawa and Zobiya, both in the Dutse local government area of Jigawa State, Nigeria. Jigawa is a State in Nothern Nigeria (Figure 1A). The state comprises 27 local government areas (LGAs) of which Dutse is one (Figure 1B). Jidawa is located at latitude 11°44’60’’N and longitude 9°15’18’’E, and Zobiya at latitude 11°44’59’’N and longitude 9°15’13’’E (Figure 1C). The two communities are drained by streams and ponds, and monthly temperatures vary between 14–38.5°C, with the maximum temperature experienced during the hottest month of April. The dry season starts early November and usually ends mid-June, and the rainy season lasts from late June to early October. The rainy season is the period for intensive agricultural activities (crops, livestock, and fishing). Lack of potable water is a major contributor to the prevalence of schistosomiasis [23]. There is no source of potable drinking water, and residents rely on the Warwade dam, an embankment near Warwade town (Figure 1C) that was built in the early 1970s. The Warwade dam area, Jigawa State, was previously reported to be a habitat for freshwater snails, including those that transmit *Schistosoma* [24].

### Study population and sampling technique

This study is a cross-sectional survey involving primary/elementary school children of grades 2–6, their parents, and classroom teachers of two elementary schools, one in each of the Jidawa and Zobiya communities. The number of pupils to be included in the study was calculated using the single proportion sample size formula with an assumption of a 95% confidence interval (CI) [25].

{\rm{n}} = \frac{{{Z^2}{\rm{ \times }}p{\rm{(1 - }}p{\rm{)}}}}{{mo{e^2}}}

Z = 1.96 (at 95% CI level)

p = 0.05 (prevalence of UgS in Jigawa State is 5% [26]

moe = 0.05 (margin of error)

{\rm{n}} = \frac{{{{1.96}^2}{\rm{ \times 0}}{\rm{.05(1 - 0}}{\rm{.05)}}}}{{{{0.05}^2}}} = 73

The estimated minimum sample size required for this study was 73 pupils from both schools. In order to make our study more robust and to accommodate participant withdrawal/dropout, we multiplied the estimate by four. Accordingly, 146 pupils were recruited by systematic random selection from each of the elementary schools at Jidawa and Zobiya, making a total of 292 pupils. The parents of the selected children were contacted to participate in focus group discussions (FGD) and to provide information on the study. Parents were also asked to participate in the survey. Signed informed consent from all the study participants (pupils, parents, and teachers) was obtained. All participants were assigned a unique study ID number.

### Socio-demographic, knowledge, attitude, and practice (KAP), and risk factor assessment

Structured questionnaires were administered verbally in the Hausa language to both parents and pupils. Information on socio-economic status and parental KAP were obtained from parents. The parent-specific questionnaire also posed questions such as level of education, vocation, and knowledge on the cause/transmission of UgS and how it can be prevented and treated. Information on demography, child KAP, and risk assessment was obtained from the pupil questionnaires. Pupils were also asked about their age, grade level, water contact activities, after school routines, and symptoms of UgS and their effects. In the questionaire (supplemental material), UgS was reffered to as bilharzia and *fitsarin tsagia* (UgS in Hausa language).

### Sample collection and parasitological analysis

Each pupil was given a sterile transparent capped urine sample container that was labelled with the same unique ID number as on the respective questionaires. The pupils were carefully instructed on how to dispense 20 mL mid-stream urine into the sample container. All collections were made in a designated area within the school premises between 1000 and 1400 h local time and processed immediately after visual inspection for the presence or absence of macro-haematuria. The timing was to ensure that the pupils had already engaged in physical activities, which promotes maximum excretion of parasite eggs [27]. Urine samples <7 mL were not used for parasitological analysis. All the children recruited for this study were treated with 40 mg/kg PZQ (Biltricide, Batch No. M94901) under the supervision of community health workers. In addition, mass administration of PZQ to children in Jidawa and Zobiya communities was suggested to the State Ministry of Health.

The urine filtration technique as recommended by the WHO for the detection of *Schistosoma haematobium* eggs in urine was used [28,29]. Briefly, 10 mL of a urine sample was aspirated into a syringe containing a few drops of Lugol’s iodine solution and agitated for 15 seconds to stain any *S. haematobium* eggs present. The sample was then gently forced through a 12-µm pore size polycarbonate membrane filter (Merck KGaA, Darmstadt, Germany) that was placed in a swinnex filter holder. Remnants of urine were removed by forcing air through the filter membrane using an empty syringe. The filter holder was unscrewed and the filter membrane removed with the aid of forceps, placed on a microscope slide, and viewed at ×400 magnification. The schistosome eggs were seen as orange oval shapped bodies, and the egg counts/10 mL of urine were documented. Egg counts were adjusted to 10 mL for urine samples ≥7 mL but <10 mL.

### Statistical analysis

The completeness of the questionaires was checked and the data were entered into Microsoft Excel and loaded into SPSS version 20.0 (IBM) for statistical analysis. The Pearson Chi-Square (χ^2^) was used to compare differences in prevalence between age groups, gender, parents’ occupation, and education level. The association between water contact activities and the prevalence of infection was determined using binary logistic regression analysis to obtain the odds ratios. Statistial significance was set at p < 0.05.

## Results

**Figure 2:**
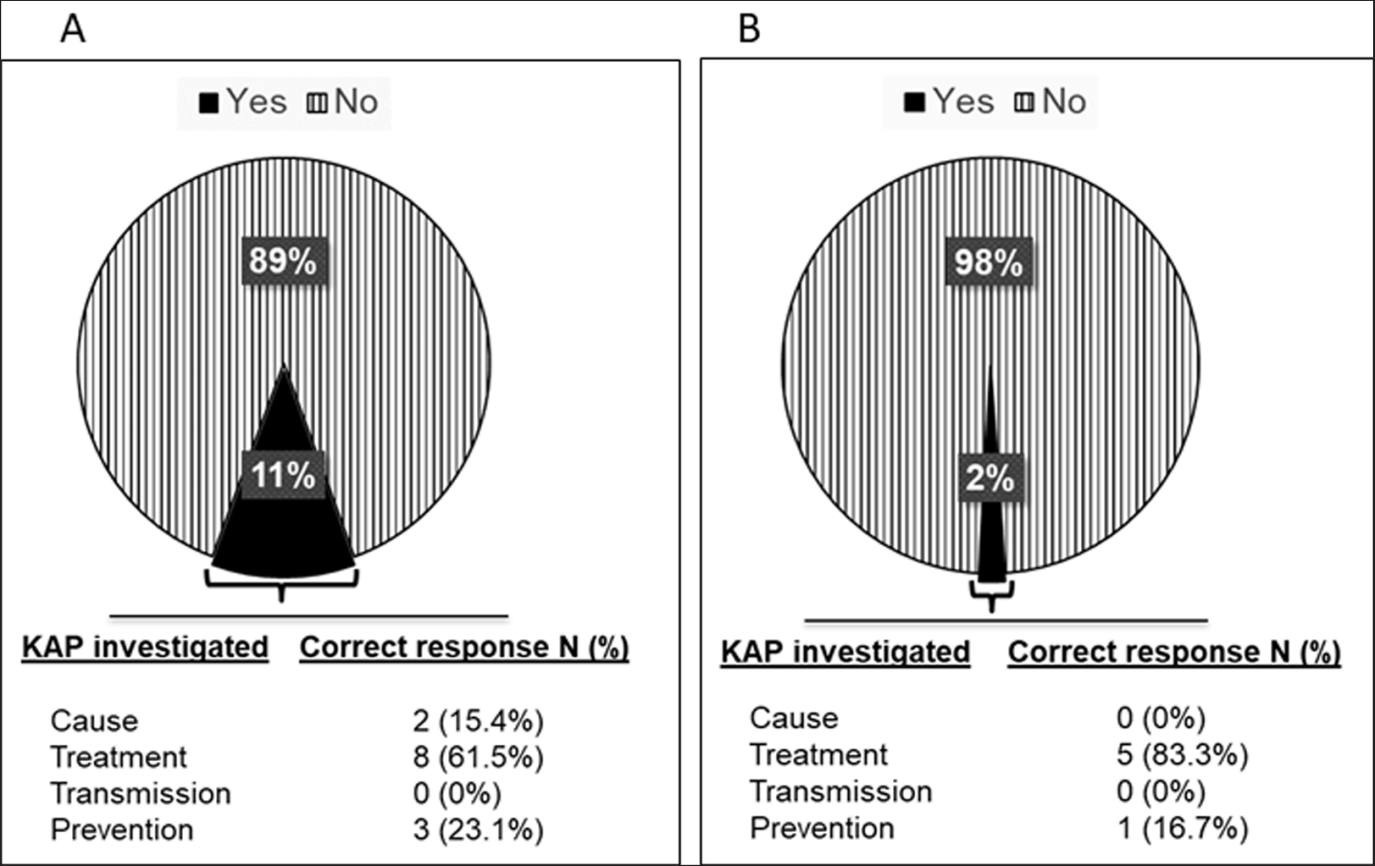
The proportion of participants with adequate knowledge of KAP on UgS. **(A)** Parents. **(B)** Children. The participants were asked in the local language via a questionnaire about knowledge of schistosomiasis (described as blood in urine), its cause, whether they know that treatment is available for it, how it is contracted/transmitted, how it can be prevented, their feelings/concerns (attitudes) towards the disease, and their water contact activities/behavior (practice). Those with one correct response were coded as Yes and those with not a single correct response as NO. The results revealed that approximately 89% and 98% of adults and children, respectively, in the study communities lack adequate KAP on UgS.

For Jidawa and Zobiya there is one elementary school in each community, and both were enrolled for this survey. A total of 292 pupils (ages 5–16 years) were selected for the study. Prior to commencement of sample collection, our team, including health care officials from the Jigawa State Ministry of Health, visited the study communities on three three occasions for the following purposes: (1) familiarisation visits to get acquainted with the location and to meet with the community heads, the head teachers, and the leaders of the parent-teacher associations, (2) community-wide sensitisation on the proposed study using public address system, (3) carrying out the FGDs and administration of questionaires. Parents provided consent, completed the questionnaire on parental KAP and socio-economic profiling, and volunteered in the FGDs. Of the 114 parents that responded, just 11.2% were partly aware of the cause of UgS, the preventive behaviours or the availability of treatment (Figure 2A). Of the 292 pupils who were given the questionnaire, 290 responded, and of these, just 2% were partially correct on the KAP for UgS (Figure 2B). The breakdown shows that among answers correctly answered, knowledge of treatment was highest (13 of 19 correct answers), followed by prevention (4/19), and cause (2/19) of UgS. None of the respondents had any knowledge of the UgS transmission cycle, and in the rare cases that the parents had any applicable knowledge, it was rarely imparted on the children. The latter observation suggests that information should not be targeted (solely) at parents and that the children should be eductated directly on the causes and prevention of UgS in school.

**Figure 3:**
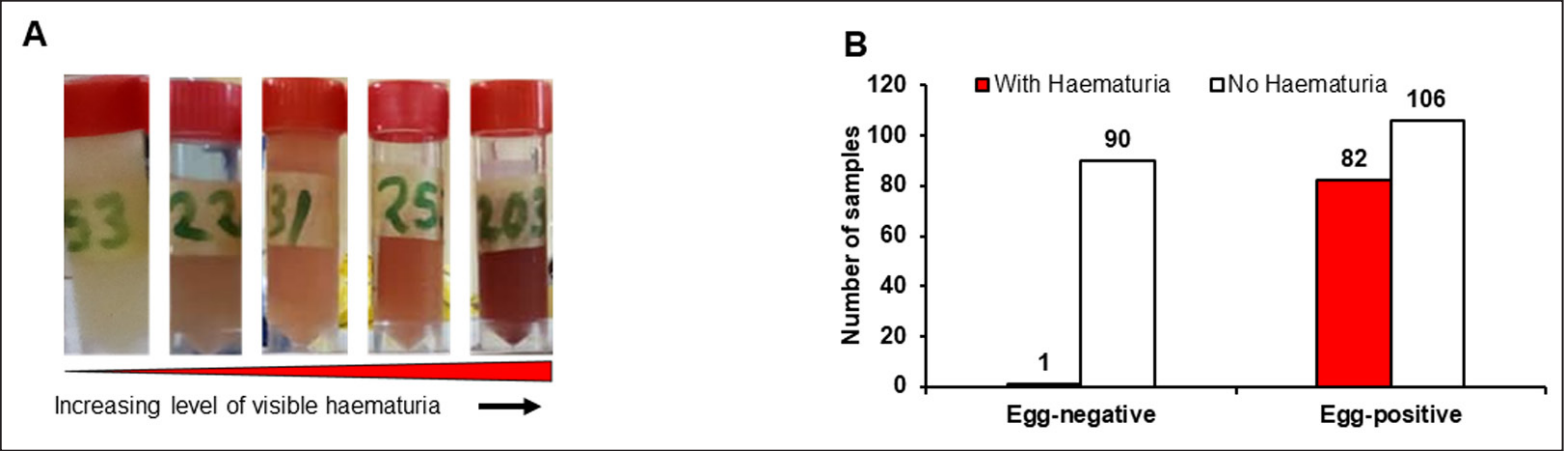
Physical analyses of urine samples. **(A)** Visual inspection showing the extent of haematuria in representative samples. **(B)** Microscopy evaluation for identification of *S. haematobium* egg-positive samples. Urine samples were separated based on the presence or absence of visible haematuria and *S. haematobium* eggs. The results show that one of the 83 (1/83) children with haematuria was apparently not due to UgS, while haematuria in the remaining 82 was contributed to by UgS. As many as 106/196 of the non-visibly haematuric samples were positive for *S. haematobium* eggs, confirming that haematuria is not a reliable diagnostic of UgS.

Of the 292 pupils enrolled, 283 provided urine samples, of which 279 samples met the criteria (urine sample volume not less than 7 mL) for analysis. Of the 279 samples, 83 showed visible/macro haematuria, and the remaining 196 appeared normal (Figure 3A). Each sample was subjected to microscopy for visualisation and counting of *S. haematobium* eggs: 188 were positive for the parasite eggs (Figure 3B), resulting in an overall prevalence of 67.3% across both communities (Jidawa and Zobiya, 69.0% and 65.7%, respectively; Table 1). Among the 188 egg-positive samples, 82 were visibly haematuric, whereas 106 were not (Figure 3B): interestingly, one of the samples with visible haematuria was devoid of *S. haematobium* eggs (Figure 3B).

**Table 1:** Relationship between demographic characteristics and behavior (water contact activity) on prevalence of urinary schistosomiasis among school-aged children in selected communities in Jigawa State, Nigeria.


LOCATION	JIDAWA (%)	ZOBIYA (%)	TOTAL (%)

Overall	90/137 (65.7)	98/142 (69.0)	188/279 (67.3)

**Age group**			

5–7	7/10 (70.0)	47/63 (74.6)	54/73 (80.0)

8–10	25/45 (55.5)	34/50 (68.0)	59/95 (62.1)

11–13	55/75 (73.3)	17/29 (58.6)	72/104 (69.2)

14–16	3/7 (42.9)	0/0 (0.0)	3/7 (42.9)

***P-value***	**0.127**	**0.300**	**0.253**

**Gender**			

Female	33/60 (55.0)	36/59 (61.0)	69/119 (58.0)

Male	57/77 (71.4)	62/83 (74.7)	119/160 (74.4)

***P-value***	**0.020**	**0.080**	**0.005**

**Grade Level**			

2	18/30 (60.0)	19/28 (67.9)	37/58 (63.8)

3	18/35 (57.4)	26/31 (83.9)	44/66 (66.7)

4	12/22 (54.5)	20/30 (66.7)	32/52 (61.5)

5	25/25 (100.0)	19/30 (63.4)	44/55 (80.0)

6	17/25 (68.0)	14/23 (60.9)	31/48 (64.6)

***P-value***	**0.001**	**0.035**	**0.003**

**Water contact activity**			

Washing	0/19 (0.0)	30/44 (68.2)	30/63 (47.6)

Irrigation farming	29/39 (74.4)	25/36 (69.4)	54/75 (72.0)

Fishing	0/0 (0.0)	4/4 (100.0)	4/4 (100.0)

Swimming	61/79 (77.2)	39/45 (86.7)	100/124 (80.6)

None	0/0 (0.0)	0/13 (0.0)	0/13 (0.0%)

***P-value***	**0.001**	**0.001**	**0.001**


**Figure 4:**
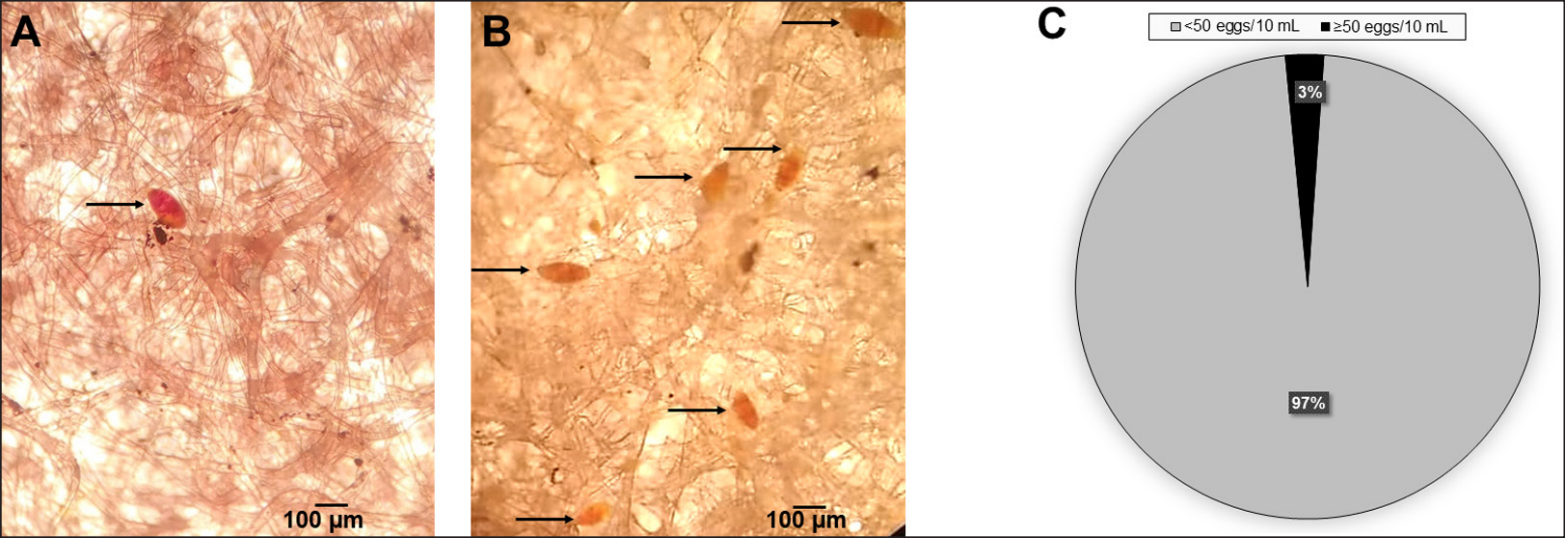
Intensity of UgS in the study communities. **(A)** and **(B)** Identification and counting of *S. haematobium* eggs in urine samples and comparison of egg density between positive urine samples with micro haematuria **(A)** and macro haematuria **(B)**. Urine samples (10 mL) were filtered through a nucleopore membrane followed by addition of Lugol’s iodine solution to the membrane. The *S. haematobium* eggs were stained, making identification and diagnosis easy. **(C)** Parasite burden was based on WHO criteria [34]: 97% and 3% of the children have moderate (<50 eggs/10 mL) and high (≥50 eggs/10 mL) intensities of infection, respectively.

The Lugol’s iodine stained the eggs and made them distinct for counting under the microscope (Figure 4A and B). The estimated dimensions of the eggs encountered were approximately 40–70 µm in width × 120–160 µm in length (Figure 4A and B) (i.e., within the expected size for *S. haematobium*) [9]. The average number of eggs in urine samples with no visible haematuria was much lower than those in haematuric urine samples (Figure 4A and 4B). In order to determine the intensity of infection in the sampled pupils based on WHO guidelines, a frequency table was constructed for the number of samples with 0, 1–49, and ≥50 eggs/10 mL of urine. The data revealed that approximately 97% had <50 eggs and 3% had ≥50 eggs (Figure 4C).

Analyses of the socio-demographic information of pupils showed that UgS is most prevalent among children of the age group 11–13 years (69.2% prevalence) in Jidawa and among children of the age group 5–7 years (74.6% prevalence) in Zobiya. With prevalence values of >50%, both communities are therefore categorised as being hyper-endemic to UgS. Overall, children aged 14–16 years had the lowest prevalence, at 42.9% (Table 1). However, statistical analysis demonstrated that there was no association between the occurrence of infection and age group (χ^2^ = 5.7, p > 0.05). Based on gender, prevalence among males and females was 74.4% and 58.0%, respectively (significant: χ^2^ = 5.4, p = 0.001; Table 1). With regard to grade level, fifth grade pupils showed the highest prevalence (80.0%); whereas, prevalence was least among fourth grade pupils (61.5%; χ^2^ = 17.92, p = 0.03; Table 1).

The prevalence of *S. haematobium* infection in relation to water contact activities among pupils was, in descending order, fishing (100.0%), swimming (80.6%), irrigation farming (72%), and washing (47.6%). Children not engaging in any of these activities had zero prevalence. There was a highly significant association between the occurrence of *S. haematobium* infection and water contact activity (χ^2^ = 38.7; p ˂ 0.05; Table 1).

Based on infection status, activities in freshwater bodies, such as swimming, washing, fishing in the resevoir, in addition to using the dam water for farm irrigation, were each associated with significant crude odds of infection with urinary schistosomiasis in Jigawa State, with the likelihood of increasing the odds of infection by 4- to 5-times in Jidawa (AOR = 5.4, 95% CI 0.99–28.86, p = 0.05) and Zobiya (AOR = 4.1, 95% CI 1.71–9.86, p = 0.05; Table 2). Urinary schistosomiasis (AOR = 1.1, 95% CI 0.44–0.26, p = 0.001) was associated with gender in Zobiya but not in Jidawa (AOR = 0.7, 95% CI 0.12–0.12, p = 0.63). Individually, people in age groups 11–13 years in Zobiya were at significantly higher crude odds of infection compared to other age groups across the two communities. In Jidawa, the age-related odds ratio are not significantly different (p = 0.07–0.69).

**Table 2:** Predisposing factors to urinary schistosomiasis in Jigawa State, Nigeria (*n* = 279).


	JIDAWA		ZOBIYA	
		
AGE GROUP	AOR, % (95% CI)	P-VALUE	AOR, % (95% CI)	P-VALUE

5–7	1.0	0.07	1.0	0.13

8–10	2.5 (1.68–4.08)	0.19	2.0 (1.26–4.21)	0.15

11–13	1.3 (0.18–3.65)	0.79	2.5 (1.03–5.14)	0.05

14–16	1.9 (0.41–9.49)	0.08	2.8 (0.20–5.55)	0.29

**Gender**				

Male	2.33 (1.13–4.79)	0.021	1.9 (0.95–4.09)	0.08

Female	1.08 (0.86–2.33)	0.27	1.1 (0.44–3.26)	0.08

**Water contact activity**	5.4 (0.99–28.9)	0.007	4.1 (1.71–9.86)	0.005


Finally, those individuals with obvious haematuria had higher infection rates (p = 0.001) with 100% and 98.1% in Jidawa and Zobiya, respectively, compared to subjects without haematuria, showing lower infection rates of 39.74 and 52.2%, respectively, in the two communities (Table 3). This implies that an average of ~99% of haematuric children in both communities were infected by *S. haematobium*, while 46% of non-visibly haematuric children were actually infected.

**Table 3:** Association between urinary schistosomiasis and haematuria among school-aged children in selected communities in Jigawa State, Nigeria.


	JIDAWA		ZOBIYA	
		
HAEMATURIA	NUMBER EXAMINED	NUMBER INFECTED (%)	NUMBER EXAMINED	NUMBER INFECTED (%)

Present	59	59 (100)	52	51 (98.1)

Absent	78	31 (39.7)	90	47 (52.2)

χ*^2^- value*		54.1		32.5

*P-value*		0.001		0.003


## Discussion

The prevalence of UgS in Nigeria is location-dependent [18,19]. In 1987, prevalence was as high as 90% in most parts of Nothern Nigeria but was negligible 500 km away in some South-Eastern states. Within Nigerian states there is also wide variation. For example, in Lagos state, prevalence was over 90% in Epe but less than 3% in Badagry [30]. Such variation makes estimating the national prevalence difficult and is compounded by the relatively slow pace of progress towards control of the disease, despite the availability of praziquantel as an effective chemotherapy [31]. It is, therefore, important to continue to expand surveillance for the disease, especially in those areas as yet unsurveyed.

Given the proximity of Jidawa and Zobiya communities to the major water resource of the Wawade dam and the lack of available data on UgS for these communities, we investigated disease prevalence in the elementary school pupils of both communities. A previous report had shown that prevalence was ~5% in Jigawa State [26], but our data indicate an approximately 13-fold higher prevalence value (67.3%; Table 1) for these two communities. This large difference is, in part, due to the fact that the previous study was hospital-based and only children attending treatment for other illnesses were recruited for the survey [26]; whereas, we perfomed community sampling, which would likely capture more people with UgS and provide the true picture of the disease in the communities.

High dropout or low response rates are a major concern in epidemiological surveys, depending on the study design, and a 70% response rate is considered desirable [32,33]. In this study, we recorded a very low number of drop-outs; only two of the 292 selected participants were unable to participate due to health reasons. Four additional participants were excluded because their urine volumes were less than 7 mL. This high response rate (~96%) is attributable to the methodical pre-survey community sensitization and public enlightenment campaigns on the purpose of the study, involving the State Ministry of Health officials, clerics, teachers, and community leaders. Even though people are aware of the disease, based on the KAP data, 89% and 98% of parents and children, respectively (Figure 2A and 2B), lack any specific knowledge on its cause, how it is contracted, the vectors, or treatment. Many survey participants view UgS as a natural/mysterious occurrence that almost every child experiences and is only curable via supernatural intervention. If our KAP data are representative of other communities, then it is recommended that state ministries of health incorporate public information regarding UgS as a key strategy towards its control and eradication in rural communities. It is clear that if (re)infection rates are to be brought under control, water-contact behaviour must change, and this is most easily achieved if both children and parents understand the risks and consequences. It could be argued that the information should be taught in shool and targeted particularly at the (somewhat older) children, who are the most at risk and will be the parents of the next generation, providing a long-term benefit by anchoring the understanding deeply into the community.

Macrohaematuria or visible blood in urine is considered a sign of UgS, most especially among school-age children [34]. In this study, just 83 (44%) of the 188 egg-positive urine samples had macrohaematuria of varying blood intensity (Figure 3A and 3B), while the remainder had no visible haematuria (Figure 3B). This observation underscores the unreliability of visual examination of urine samples as a diagnostic approach in the field. However, of those samples that were visibly haematuric, approximately 99% were egg-positive. Importantly, the egg counts in the urine samples with macrohaematuria were higher than in the urine samples without visible haematuria (Figure 4A and 4B). The implication of this is that in endemic regions it is fair to conclude that the presence of blood in urine of children serves as strong prognosis of UgS. Most of the infected children (97%) in the Jidawa and Zobiya communities had 20–48 eggs/10 mL of urine, whereas 3% showed ≥50 egg counts (Figure 4C), indicating moderate and high intensity of infection in these communities, respectively, based on the WHO classification [35]. In contrast to the samples with egg counts of ≥50/10 mL, which were all (100%) macrohaematuric, only 42% of samples with <50 eggs/10 mL were macrohaematuric. This observation clearly supports the Bayesian inference on hematuria and egg intensity in urine of individuals living in schistosomiasis-endemic foci [36]. A single participant, a 10-year-old male in Zobiya community, presented with macrohaematuria but was negative for *S. haematobium* eggs (Figure 3B). The cause of haematuria in this case may be due to other ailments, such as a bacterial infection or kidney disease. His family was advised to consult a physician.

The prevalence rate of infection of 67.3% determined in this study falls within the WHO classification ‘hyperendemic’ [35]. Prevalence values of 1–10%, 11–50%, and >50% are regarded as low, moderately, and hyper-endemic, respectively. The two communities have prevalences far greater than those determined earlier in Osun state (12.7%) [37], Benue State (42.6% in both Ogbadibo [38] and Katsina LGAs [39,40]), and the Ipogun community of Ondo State (24.9%) [41]. Our prevalence data are more in line with the findings of other studies, which reported high prevalences of urinary schistosomiasis in rural areas of Nigeria: 75.6% in Ogbesi-Ekiti, Ekiti State [42] and 72.0% in Dutsinma, Katsina State [43].

The high rate of infection reported in the present study may be an indication of the rate of *S. haematobium* transmission in and around the Warwade dam and local streams. Locals depend on these water bodies for fishing, bathing, swimming, and other domestic needs, and the water contact provides opportunities for infection and reinfection. The infection pattern noted here showed a typical gender-related prevalence whereby males were infected more often than females [21,41,44]. This could be due to the higher tendencies of water contact activities among males through swimming, playing, and laundry in addition to the domestic activity of fetching water, which exposes both sexes to infection. Similar trends have been recorded in other endemic settings, both in Nigeria and elsewhere in Africa [12,45], while other studies have found no significant differences in gender prevalence [46,47]. Of note here, 13 children reported no water contact activities at all. The data revealed that this group of children are from homes where parents have some KAP for UgS (i.e., are aware of the cause and of preventive steps) and are mostly civil servants or businessmen. In addition, eight of these 13 children were familiar with the potential dangers of swimming by the dam. This observation illustrates the potential benefits of public education on the health implications of water contact regarding schistosomiasis.

UgS is most prevalent (80%) among children 5–7 years old in both Jidawa and Zobiya communities (Table 1). This is because most of the children are already engaged in water contact activities as early as pre-school age (5 years old and below), meaning that they are likely to have been exposed and infected before school enrolment. At the moment, state-level efforts on the mass administration of praziquantel mostly targets school-aged children. Our data is suggesting the need for the expansion of this policy to mandatorily include pre-school children. Reports on UgS amongst pre-school are still very scant, but the few available have painted an alarming picture. For instance, the prevalence of pre-school (<5 years old) UgS is as high as 60% in Falmado, Niger Republic [48] and 71% in Oyan, Nigeria [49]. Owing to our speculation on the high prevalence of pre-school UgS in the study location, we plan to next conduct a survey of UgS amongst pre-schoolers in Jidawa and Zobiya communities. Finally, as a result of climate change and the consequent expanding habitats for vectors of infectious diseases [50], continued surveillance is highly recommended.

## Conclusion

We have conducted the first community-based survey of UgS in Jigawa State, Nigeria. The results reveal that the study communities, Jidawa and Zobiya of Dutse LGA, are hyperendemic for UgS. The findings herein highlight the need for similar studies to be conducted in other communities in the state and nationally in order to have more accurate data with higher granularity, and a better plan for treatment and control programs. It should be noted that vocations such as fishing and irrigation farming were found to be important risk factors for UgS, but even more important is that we documented an alarmingly poor level of KAP on schistosomiasis (lack of knowledge on UgS cause, treatment, prevention, and risky activities such as swimming near the dam) among the community residents (children, parents, teachers, and community health workers). In these situaitons, it is recommended that local and state authorities include information and education as key components of any intervention stategy. Finally, after this study, we have collaborated with State Ministry officials to carry out a mass administration of praziquantel to the school children in Jidawa and Zobiya communities in April 2021.

## Institutional Review Board Statement

The study was conducted according to the guidelines of the Declaration of Helsinki, approved by the Institutional Review Board (or Ethics Committee) of Federal University Dutse and Ministry of Health Jigawa State, Nigeria (Ref. No.: MOH/Sec/1.S/528/Vol. 1) on February 27, 2020.

## Data Accessibility Statement

The data presented in this study are available on request from the corresponding author. The data are not publicly available due to ethical considerations.
